# Elevated endoplasmic reticulum stress reinforced immunosuppression in the tumor microenvironment *via* myeloid-derived suppressor cells

**DOI:** 10.18632/oncotarget.2589

**Published:** 2014-12-02

**Authors:** Bo-Ra Lee, Sun-Young Chang, Eun-Hye Hong, Bo-Eun Kwon, Hong Min Kim, Yeon-Jeong Kim, Jongkook Lee, Hyun-Jong Cho, Jae-Hee Cheon, Hyun-Jeong Ko

**Affiliations:** ^1^ College of Pharmacy, Kangwon National University, Chuncheon 200-701, Korea; ^2^ College of Pharmacy, Ajou University, Suwon 443-749, Korea; ^3^ Department of Internal Medicine, Yonsei University Wonju College of Medicine, Wonju 220-701, Korea; ^4^ College of Pharmacy, Inje University, Gimhae 621-749, Korea; ^5^ Department of Internal Medicine and Institute of Gastroenterology, Yonsei University College of Medicine, Seoul 120-752, Korea

**Keywords:** cancer, ER stress, MDSC, immunosuppression, arginase-1

## Abstract

The role of endoplasmic reticulum (ER) stress in cancer has been studied in detail, and ER stress is known to increase tumor cell apoptosis, and thus, reduce tumor growth. However, in our study, persistent ER stress induced by multiple administrations of low-dose thapsigargin (Tg) accelerated tumor growth in mice. Tg-mediated ER stress increased the generation of Ly6G^+^CD11b^+^ myeloid cells, but did not alter anti-tumor effector T cells. 4-Phenylbutyric acid (4-PBA), a chemical chaperone widely used as an ER stress reducer, attenuated Tg-induced myeloid-derived suppressor cell (MDSC) expansion and tumor growth. Tg-mediated ER stress enhanced the immunosuppressive capacity of tumor-infiltrating MDSCs by increasing expression of ARG1, iNOS, and NOX2, although splenic MDSCs were not affected. Consistent with these results, 4-PBA restored the anti-tumor immune response by regulating inflammatory cytokines such as TNF-α and CXCL1/KC, and activated tumor-infiltrating CD8^+^ T cells that were inhibited by Tg-mediated ER stress. These results suggest that significant ER stress in a tumor-bearing host might induce tumor growth mediated by enhancement of MDSC-mediated suppression. Therefore, ER stress reducers such as 4-PBA could restore anti-tumor immunity by inhibiting suppressive MDSCs that are exacerbated by ER stress.

## INTRODUCTION

Endoplasmic reticulum (ER) stress induction in cancer cells was once a promising strategy for efficiently inducing apoptosis of cancer cells, and several ER stress inducers, including thapsigargin (Tg), a sarcoplasmic/endoplasmic Ca^2+^ ATPase inhibitor, were evaluated as anticancer drugs [1]. However, systemic administration of Tg induced non-selective apoptosis of host cells, including proliferative and quiescent cells [2], indicating that Tg might not be a good candidate anticancer drug for systemic administration. To overcome this limitation, targeted delivery of Tg has been evaluated in several murine cancer models [1, 3].

Although direct induction of apoptotic death in cancer cells may be critical for therapeutic antitumor effects, the tumor antigen-specific immune response against cancer cells has also been shown to efficiently inhibit tumor growth and eradicate residual tumor cells. In this regard, Tg-induced cell death could beneficially induce tumor-specific immunity following phagocytosis by dendritic cells [1]. We as well as other researchers have provided evidence supporting the combined therapeutic strategy of apoptosis induction by anticancer drugs and activation of the host immune system to remove residual cancer cells [4].

Myeloid-derived suppressor cells (MDSCs) are heterogeneous cell populations consisting of immature myeloid cells including immature macrophages, granulocytes, and dendritic cells. They are found in the blood, liver, and spleen, as well as in tumor cells [5, 6]. More importantly, it has been suggested that tumor-associated suppressor cells, including MDSCs, inhibit the tumor-specific immune response, and inflammation caused by non-selective ER stress-mediated cell death could be associated with the induction of MDSC-like cells [7].

Here we investigated the effect of ER stress induced by treatment with Tg, an ER stress inducer, on tumor growth. Surprisingly, systemic administration of Tg did not inhibit tumor growth, but instead, increased tumor growth. Although the role of ER stress in cancer has been studied in detail, the effects of ER stress on tumor-associated immune cells, especially MDSCs, have not been reported. Thus, we studied the effect of ER stress on MDSC generation after Tg administration *in vivo*. Interestingly, persistent and systemic ER stress induced by low dose and long-term administration of Tg increased MDSC generation in the spleens of tumor-bearing mice. More importantly, the immunosuppressive function of MDSCs, which is mediated by ARG1, iNOS, and NOX2, was also significantly increased by Tg treatment. Alleviation of ER stress by the administration of 4-PBA, a chemical chaperone used as an ER stress reducer [8], effectively restored the level of MDSCs and ameliorated their suppressive function in the tumor tissues, but had no effect on MDSCs in the spleen. Consistent with these results, accelerated tumor growth triggered by Tg was inhibited by 4-PBA treatment. These results suggest that ER stress in a tumor-bearing host might induce tumor growth by reinforcing the suppressive function of MDSCs within the tumor microenvironment.

## RESULTS

### Tg treatment accelerated tumor growth in a mouse colon cancer model

**Figure 1:**
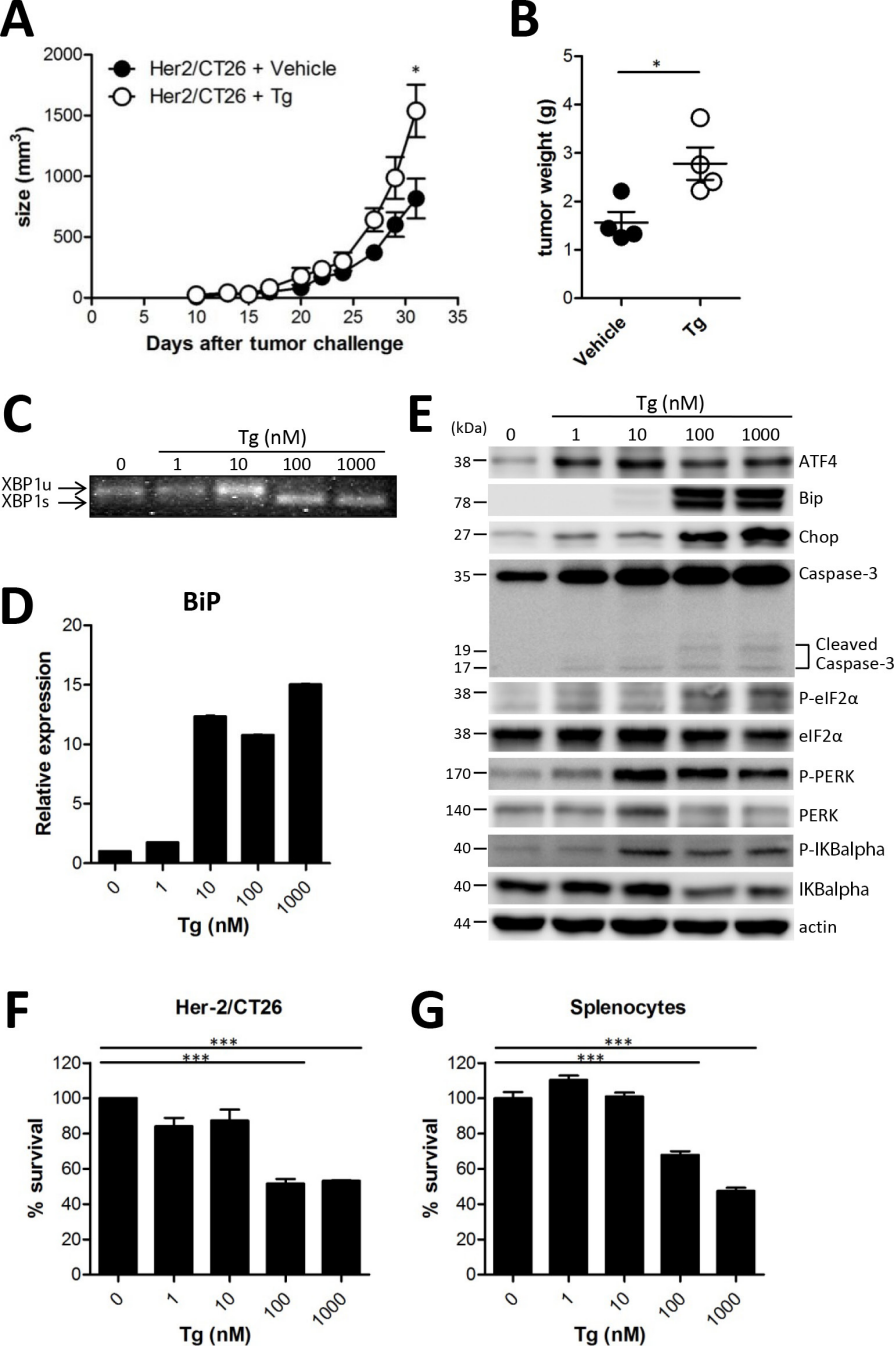
ER stress induced by Tg accelerated tumor growth **(A)** BALB/c mice were injected *s.c.* with 10^6^ HER2/CT26 cells per mouse, and 100 μg/kg of Tg was administered *i.p.* every day before tumor challenge. Tumor growth was monitored (*n* = 5). **(B)** tumor weight at 4 weeks after HER2/CT26 injection (*n* = 4). Graphs show mean ± SEM. ^*^p < 0.05, ^***^p < 0.001 compared with matched control group using the *Student's t-test*. **(C)** *XBP1* mRNA splicing in Tg-treated HER2/CT26 cells. **(D)** mRNA levels of BiP from Tg-treated HER2/CT26 cells as measured by RT-qPCR. **(E)** immunoblot of Tg-treated HER2/CT26 cells for the PERK-eIF2α branch. **(F)** HER2/CT26 cells were cultured with Tg for 24 h and cell viability was analyzed. **(G)** splenocytes were cultured with Tg for 24 h and cell viability was analyzed. ^***^p < 0.001 using one-way ANOVA with Tukey's post hoc test.

Previously, it was reported that induction of ER stress increased apoptosis of tumor cells *in vitro*, and therefore, reduced tumor growth *in vivo* [9]. Tg is a well-known ER stress inducer that inhibits sarcoplasmic/endoplasmic Ca^2+^-ATPase [1, 10]. Although Tg induces apoptosis in both proliferative and quiescent cells, it cannot be administered systemically because of host toxicity that is related to its non-selectivity [11]. However, several reports have shown a direct anticancer activity *in vivo* using a targeted Tg delivery system [3, 11]. Thus, we assessed the anticancer activity of Tg in a murine colon cancer model. To reduce systemic and acute host toxicity, we adopted a low dose and long-term Tg treatment regimen. Groups of mice were subcutaneously administered 1 × 10^6^ CT26 cells expressing HER2/*neu* (HER2/CT26 cells), and 100 μg/kg of Tg was intraperitoneally injected daily starting when tumor sizes reached 50-100 mm^3^. To our surprise, tumor growth was significantly increased in mice treated with Tg as compared with mice treated with the vehicle control (Figure 1A). When isolated tumor masses were analyzed, Tg treatment was significantly associated with increased tumor weight (Figure 1B). These results suggest that ER stress induction by systemic low dose Tg treatment can enhance tumor growth *in vivo*.

### Tg evoked ER stress and cell death in HER2/CT26 cells *in vitro*

To verify ER stress in tumor and immune cells in the tumor microenvironment, we assessed the ER stress response and the subsequent cell death in HER2/CT26 cells and splenocytes 24 h after Tg treatment. We first confirmed the influence of Tg treatment on the ER stress response in HER2/CT26 tumor cells by measuring ER stress-induced *XBP1* mRNA splicing [12]. *XBP1* slicing was detected when HER2/CT26 cells were treated with 100 nM Tg (Figure 1C). Tg treatment mediated the ER stress response by transcriptional activation of several ER stress-related genes, including BiP, TNF-α, Erdj4, CXCL1, and ATF4 (Figure 1D, Supplementary Figure 1).

Activation of the PERK-eIF2α pathway is another characteristic of the ER stress response. Tg treatment increased protein expression of ATF4 and Chop and phosphorylation of eIF2α, PERK, and IKBalpha, suggesting activation of the PERK-eIF2α pathway (Figure 1E). These results clearly show that Tg treatment induced ER stress in HER2/CT26 cells. We then evaluated whether ER stress induced by Tg treatment influences cell death. Tg treatment at concentrations lower than 10 nM did not reduce cell viability in HER2/CT26 cells or splenocytes (Figures 1F and 1G). However, concentrations of Tg greater than 100 nM induced significant cell death in HER2/CT26 cells and splenocytes.

These results suggest that Tg treatment at high concentrations induces ER stress and cell death. In contrast, 10 nM Tg induced the ER stress response and increased transcriptional activation of Bip, protein expression of ATF4, and phosphorylation of PERK without inducing significant cytotoxicity. However, considered together with tumor growth observations, these effects could not explain the enhanced tumor growth cause by Tg treatment.

### Tg-mediated ER stress did not decrease the generation of antitumor effector T cells

**Figure 2:**
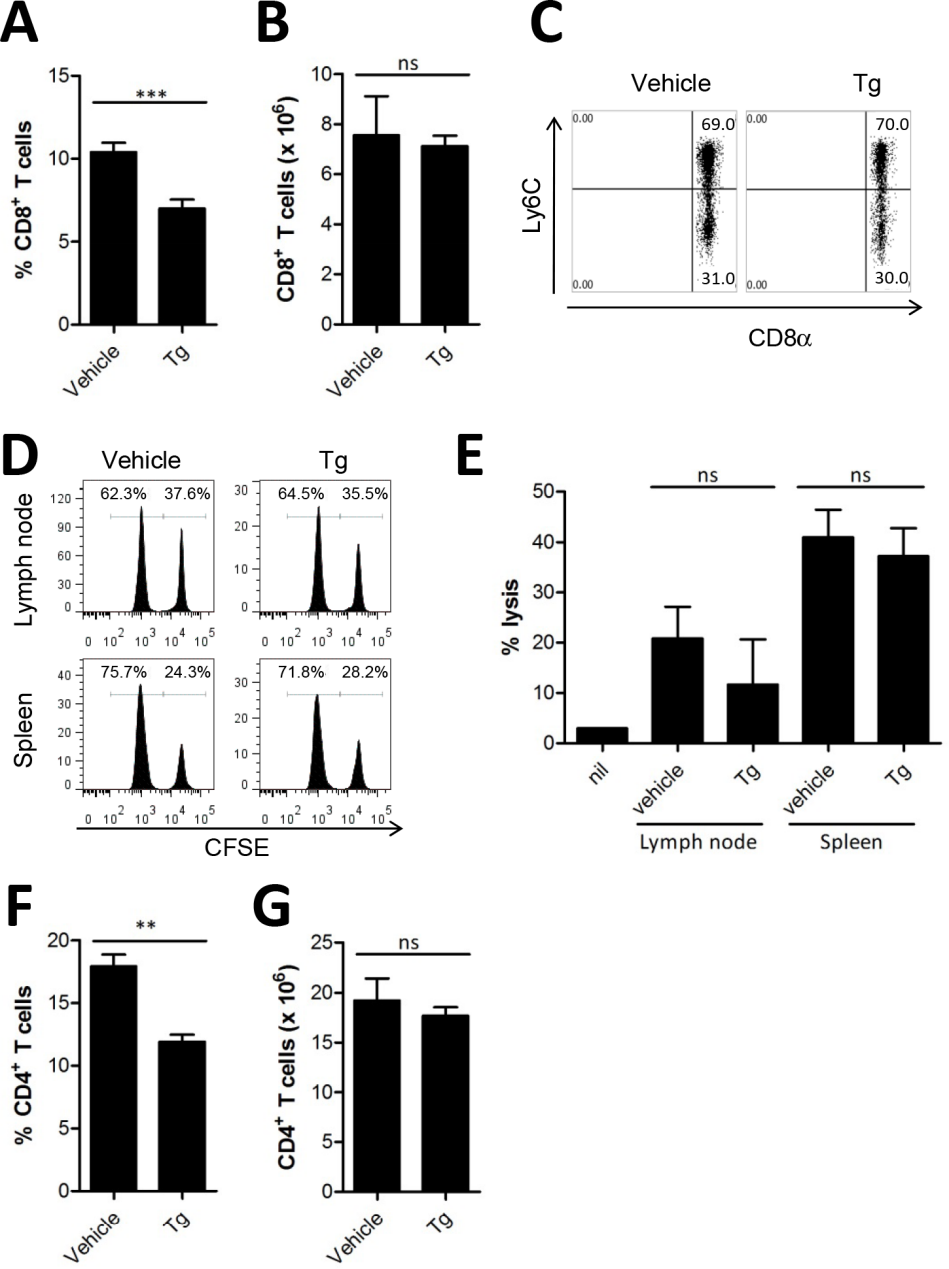
Tg-mediated ER stress did not alter anti-tumor effector T cells **(A-B)**, at 4 weeks after tumor challenge, the percentage and absolute number of CD8^+^ T cells in the spleen was assessed (*n* = 6). **(C)** percentages of Ly6C^+^ CD8^+^ T cells in the splenocytes of HER2/CT26 tumor-bearing mice. **(D-E)** specific lysis of hP63 (TYLPTNASL) peptide-loaded CFSE^high^ target cells was estimated by *in vivo* CTL from the spleen and lymph node of HER2/CT26 tumor-bearing mice (*n* = 6). **(F-G)** the percentage and absolute number of CD4^+^ T cells in the spleen of HER2/CT26-bearing mice (*n* = 7). Graphs show mean ±SEM. ns, not significant, ^**^p < 0.01; ^***^p < 0.001 compared with matched control group using the *Student's* t*-*test.

Since Tg treatment significantly increased tumor growth *in vivo* (Figure 1A and 1B), we hypothesized that Tg might negatively influence the host immune system to reduce tumor protection. Therefore, we analyzed CD8^+^ T cells, which are critical for the cytolytic elimination of cancer cells expressing tumor antigens. Although there was a significant decrease in the percentage of CD8^+^ T cells in the spleens of mice treated with Tg compared with vehicle-treated control mice (Figure 2A), the absolute number of splenic CD8^+^ T cells was not changed (Figure 2B). In addition, memory CD8^+^ T cells expressing Ly6C, which are critical for successful antitumor activity [13], were not altered (Figure 2C). Next, we assessed whether Tg treatment reduces the tumor antigen-specific CTL response by *in vivo* CTL analysis. There was no significant decrease in the HER2/*neu*-specific CTL response after Tg treatment in tumor-draining LNs or in the spleen (Figures 2D and 2E). We also examined the population of CD4^+^ T cells. Similar to CD8^+^ T cells, the percentage of CD4^+^ T cells was decreased (Figure 2F), but there was no significant decrease in the number of CD4^+^ cells after Tg treatment (Figure 2G). These results indicate that the acceleration of tumor growth produced by Tg treatment might not be attributed to inhibition of antitumor effector T cells. In addition, there appeared to be no significant systemic impairment in the tumor antigen-specific cytotoxic activity of CD8^+^ T cells after Tg treatment.

### Tg-mediated ER stress increased Ly6G^+^CD11b^+^ myeloid cells

**Figure 3:**
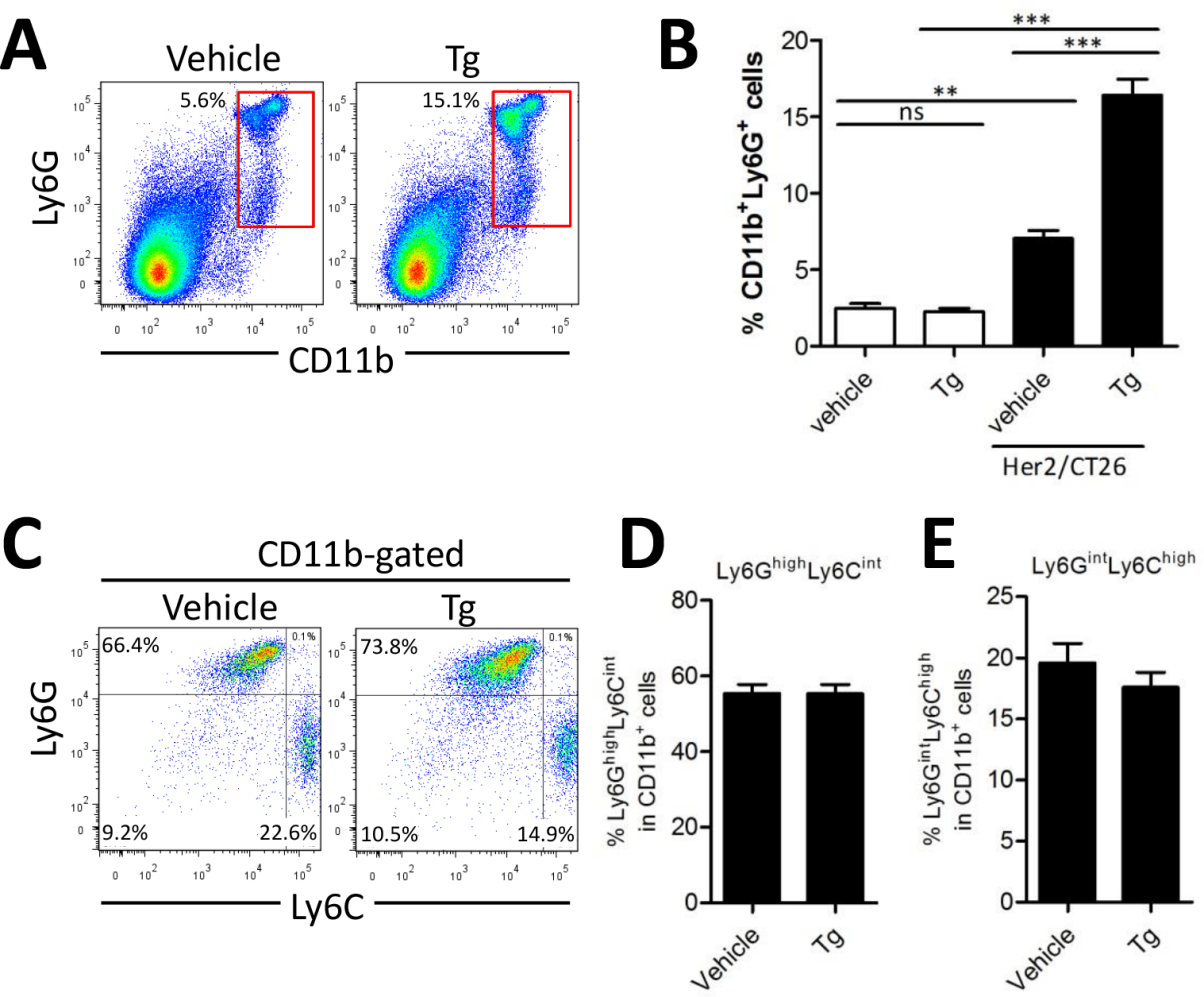
Tg-mediated ER stress increased the generation of Ly6G^+^CD11b^+^ myeloid cells HER2/CT26 tumor-bearing mice were treated with 100 μg/kg Tg. At 4 weeks after tumor injection, splenic MDSC populations were analyzed. **(A)** splenic Ly6G^+^CD11b^+^ MDSCs. **(B)** summary graph showing the frequency of Ly6G^+^CD11b^+^ cells (*n* = 6). ns, not significant, ^**^p < 0.01, ^***^p < 0.001 compared with matched control group using one-way ANOVA. **(C)** MDSC sub-populations, monocyte-derived Ly6C^high^Ly6G^int^ and granulocyte-derived Ly6C^int^Ly6G^high^ among the CD11b^+^ gated population. **(D-E)** summary graph of Ly6C^high^Ly6G^int^ and Ly6C^int^Ly6G^high^ cells among the CD11b^+^ myeloid cells (*n* = 4).

Because percentages of both CD4^+^ and CD8^+^ T cells were decreased in mice with larger tumor mass after Tg treatment, we hypothesized that immunosuppressive myeloid cells might have been expanded. Tg treatment significantly increased Ly6G^+^CD11b^+^ MDSCs in the spleen of HER2/CT26-bearing mice (16.4 ± 2.58%), as compared with vehicle control mice (7.0 ± 1.28%, Figure 3A and 3B). In the absence of tumor, percentages of splenic Ly6G^+^CD11b^+^ MDSCs were low regardless of Tg treatment. Previously, it was reported that Ly6G^+^CD11b^+^ MDSCs consisted of more than 2 major subsets, including monocytic Ly6C^high^ Ly6G^intermediate^ CD11b^+^ and granulocytic Ly6C^intermediate^ Ly6G^high^ CD11b^+^ cells. Thus, we investigated changes in the subpopulations of CD11b^+^ MDSCs in mice after Tg treatment. In spite of the expansion of Ly6G^+^CD11b^+^ MDSCs, the relative abundance of each population was not significantly changed by Tg treatment (Figures 3C–E). These results suggest that Tg treatment might enhance tumor growth via modulation of MDSCs.

### 4-PBA attenuated ER stress-induced accelerated tumor growth

**Figure 4:**
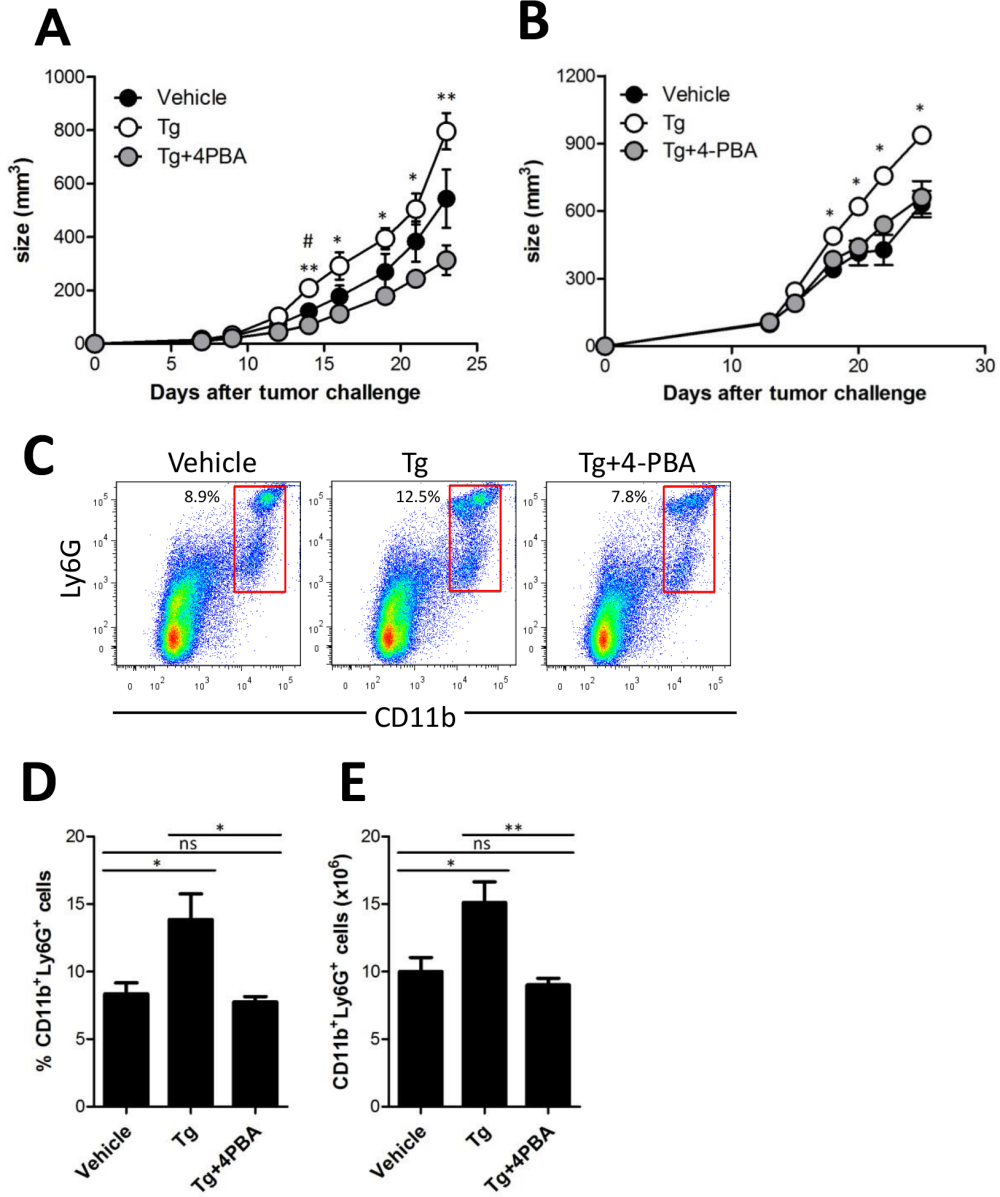
(4-PBA) attenuated Tg-induced MDSC expansion and tumor growth **(A)** two weeks after HER2/CT26 tumor inoculation, 100 μg/kg Tg or 10 mg/kg 4-PBA was administered every day. Growth of HER2/CT26 tumors were measured (*n* = 6). ^*^p < 0.05, ^**^p < 0.01 for Tg vs. Tg+4-PBA, ^#^*p* < 0.05 for vehicle vs. Tg, using one-way ANOVA. **(B)** two weeks after TC-1 tumor inoculation in C57BL/6 mice, 100 μg/kg Tg or 10 mg/kg 4-PBA was administered every day. Growth of TC-1 tumors were measured (*n* = 6). ^*^p < 0.05 using one-way ANOVA. **(C)** Ly6G^+^CD11b^+^ cells in the spleen of HER2/CT26 tumor-bearing mice treated with Tg and/or 4-PBA. **(D and E)** percentage and absolute number of Ly6G^+^CD11b^+^ MDSCs in the spleen of HER2/CT26 tumor-bearing mice treated with Tg and/or 4-PBA (*n* = 6). ns, not significant, ^*^p < 0.05, ^**^p <0.01 compared with matched control group using one-way ANOVA.

Tumor ER stress was recently suggested to be linked to the generation of tumor-infiltrating inflammatory myeloid cells [14]. Thus, we hypothesized that the increased ER stress in the tumor microenvironment could lead to induction of MDSCs *in vivo*, and therefore mediate tumor growth enhancement. To test this hypothesis, 4-PBA was used to attenuate Tg-induced ER stress in tumor-bearing mice. 4-PBA is a chemical chaperone that can decrease the ER stress response and act as a histone deacetylase inhibitor [8]. When the growth of HER2/CT26 tumors was monitored, we found that the increased tumor growth induced by Tg treatment was dramatically inhibited by coadministration of 4-PBA (Figure 4A). In another tumor model using TC-1 cells, Tg-induced tumor growth was also significantly inhibited by coadministration of 4-PBA (Figure 4B). In addition, the survival of Tg-treated mice was significantly increased by 4-PBA, compared to vehicle treatment group (Supplementary Figure 2).

To assess the potential of 4-PBA to reduce MDSC generation induced by ER stress, we examined splenic MDSCs. Treatment with Tg significantly increased Ly6G^+^CD11b^+^ MDSCs in the spleen, and coadministration of 4-PBA significantly reduced the percentage and absolute number of Ly6G^+^CD11b^+^ MDSCs, as compared with those of Tg-treated tumor-bearing mice (Figure 4C-E). However, in contrast to the results in splenic MDSCs, when tumor-infiltrating MDSCs were examined in tumor-bearing mice, we found that neither the percentage nor the absolute number of Ly6G^+^ MDSCs was increased (Supplementary Figures 3A-C). The subpopulation of CD11b^+^ MDSCs, including Ly6G^high^ Ly6C^int^ and Ly6G^int^ Ly6C^high^ cells, showed a similar profile, except for an increased Ly6G^high^ Ly6C^high^ population in the presence of ER stress (Supplementary Figure 3D-G). These results suggest that 4-PBA mitigates ER stress in the tumor microenvironment, leading to reduced tumor growth, and inhibits the expansion of splenic, but not tumor-infiltrating, Ly6G^+^CD11b^+^ MDSCs.

### ER stress reinforced immunosuppressive function of MDSCs within tumor microenvironment

**Figure 5:**
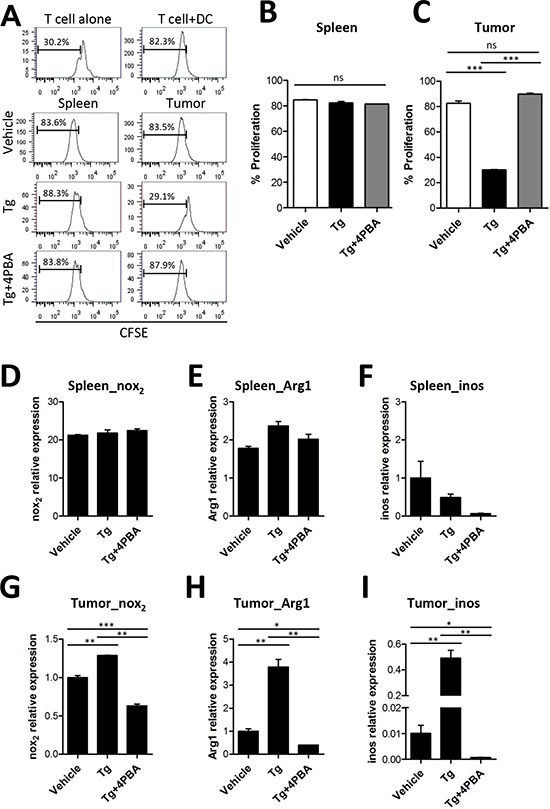
Tg-mediated ER stress reinforced the immunosuppressive capacity of tumor-infiltrating MDSCs **(A)** MDSCs were isolated from the spleen or tumors of TC-1 tumor-bearing mice. OT-II peptide-pulsed splenic DCs and CFSE-labeled OT-II CD4^+^ T cells were cocultured with MDSC for 72 h. The percentages of proliferated OT-II CD4^+^ T cells were determined *via* analysis of CFSE dilution. **(B)** proliferation of OT-II cells cocultured with spleen-infiltrating MDSCs (*n* = 3). **(C)** proliferation of OT-II cells cocultured with tumor-infiltrating MDSCs (*n* = 3). **(D-F)** mRNA levels of *Nox2*, *Arg1*, and *iNOS* in spleen-infiltrating MDSCs. **(G-I)** mRNA levels of *Nox2*, *Arg1*, and *iNOS* in tumor-infiltrating MDSCs. ns, not significant, ^*^p < 0.05, ^**^p < 0.01, ^***^p < 0.001 compared with matched control group using one-way ANOVA.

MDSCs are known as tumor-associated suppressor cells and produce immunosuppression in opposition to various types of immune cells, including CD4^+^ T cell, CD8^+^ T cell, and NK cells [15, 16]. Thus, we compared the suppressive function of MDSCs in the spleen and in the tumor tissue after Tg and/or 4-PBA treatment. MDSCs isolated from the spleen or from tumors were cocultured with OT-II peptide-pulsed splenic DCs and CFSE-labeled OT-II CD4^+^ T cells to determine T cell activation status. Despite the increased number of MDSCs in the spleen, we found no differences in the suppressive function of splenic MDSCs among vehicle, Tg, and Tg/4-PBA-treated mice (Figure 5A and 5B). In contrast, tumor-infiltrating MDSCs showed significantly increased immunosuppressive function after Tg treatment, as compared with vehicle treatment (Figure 5A and 5C). Treatment with 4-PBA significantly inhibited the suppressive function of tumor-infiltrating MDSCs under ER stress conditions (Figures 5A and 5C).

Several previous studies suggested that the immunosuppressive function of MDSCs was partly mediated by arginase-1 (ARG1) [17], iNOS [17], and reactive oxygen species (ROS) generated by NOX2 [6, 15, 18]. Therefore, we assessed the transcription levels of *ARG1*, *iNOS*, and *NOX2*. Although there were no significant changes in the mRNA levels of *NOX2* and *ARG1* in splenic MDSCs among groups, the expression levels of *NOX2*, *ARG1*, and *iNOS* were significantly increased in tumor-infiltrating MDSCs after Tg treatment, as compared with the vehicle control group (Figures 5D-I). Surprisingly, Tg-induced up-regulation of *ARG1*, *Nox2*, and *iNOS* in tumor-infiltrating MDSCs was significantly inhibited by 4-PBA treatment (Figure 5G-I). These results suggest that Tg-mediated ER stress enhances the immunosuppressive capacity of MDSCs at the local tumor site, which was mitigated by 4-PBA treatment.

### 4-PBA restored anti-tumor immunity *via* dampening suppressive MDSCs exacerbated by ER stress

Previously, we reported the conversion of immunosuppressive MDSCs into immunogenic Ag-presenting cells (APCs) using activated invariant NKT cells, and that immunogenic APCs converted from MDSCs expressed significantly increased levels of costimulatory molecules, including CD40 and CD86 [5]. When we measured the expression of costimulatory molecules in MDSCs, CD40 and CD86 expression on splenic MDSCs was not changed by treatment with Tg or 4-PBA (Supplementary Figure 4).

To identify factors associated with the Tg-mediated increased immunosuppressive function of tumor-resident MDSCs, we first assessed levels of hypoxia inducible factors (HIFs), which have been shown to induce tumor angiogenesis and to be increased in the tumor microenvironment [19]. Increased levels of HIFα proteins activate PI3K-AKT-mTOR-STAT3 signaling [20] and thus could enhance the immunosuppressive function of MDSCs in tumors. We found that Tg-mediated ER stress induced expression of HIF transcription factors (Supplementary Figure 5). Tg-mediated increases in HIF-1α and HIF-2α in the tumor microenvironment were attenuated by 4-PBA.

**Figure 6:**
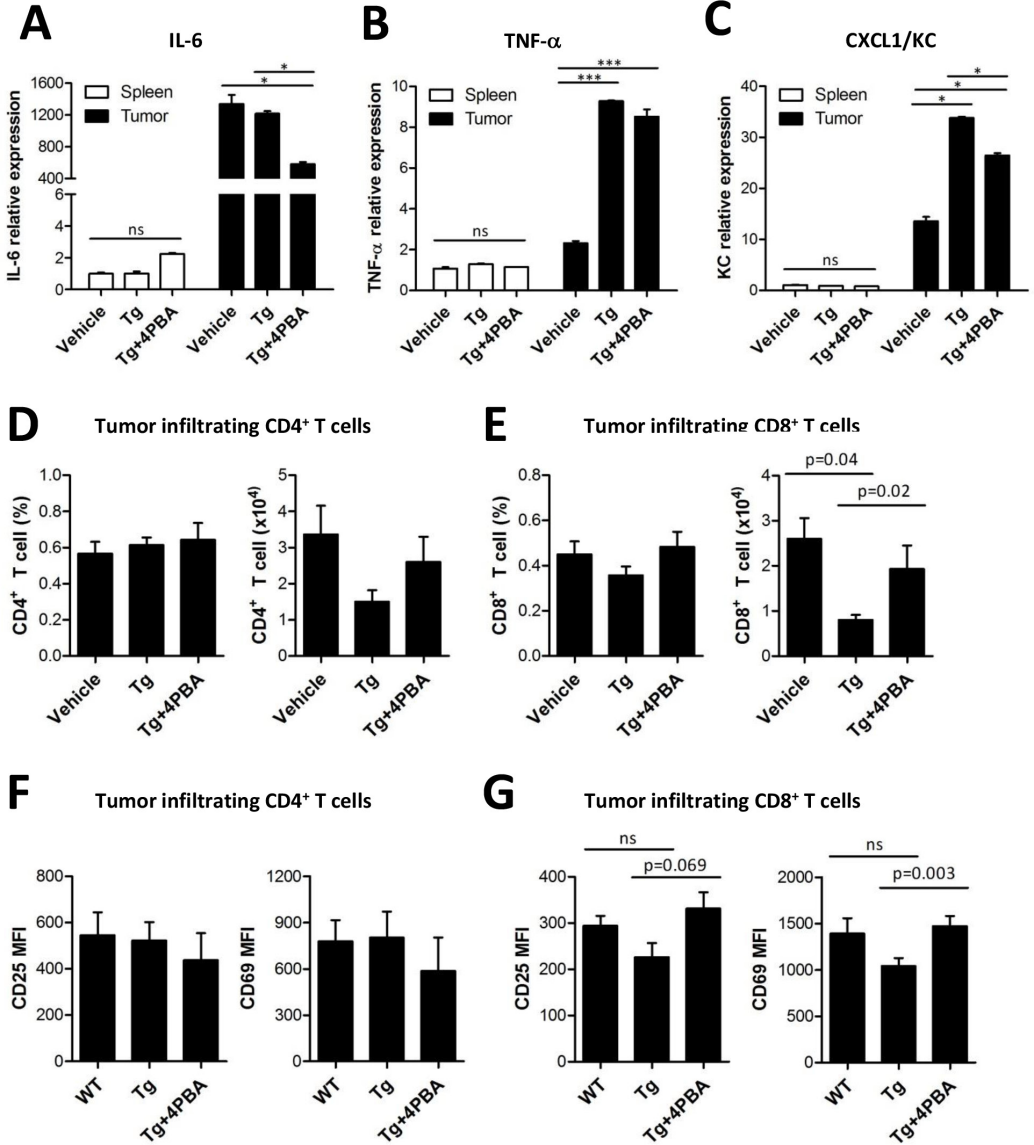
4-PBA restored anti-tumor immunity *via* dampening of suppressive MDSCs exacerbated by ER stress **(A-C)** mRNA levels of inflammatory cytokines (IL-6, TNF-α, and KC) from spleen- or tumor-filtrating MDSCs. ns, not significant, *p < 0.05, ***p < 0.001 using one-way ANOVA. **(D)** the percentage and absolute number of tumor-infiltrating CD4^+^ T cells (*n* = 3). **(E)** the percentage and absolute number of tumor-infiltrating CD8^+^ T cells (*n* = 3, one-way ANOVA). **(F)** surface expression of activation markers CD25 and CD69 in tumor-infiltrating CD4^+^ T cells (*n* = 3). **(G)** surface expression of activation markers CD25 and CD69 in tumor-infiltrating CD8^+^ T cells (*n* = 3, one-way ANOVA).

In addition, NF-κB-dependent inflammatory cytokines such as IL-6, TNF-α, and CXCL1/KC have been linked with the enhanced immunosuppressive activity of tumor-infiltrating MDSCs [14, 21]. Expression of these proinflammatory cytokines was enhanced by Tg-mediated ER stress, and this increased expression was attenuated by 4-PBA treatment (Figure 6A-C). Finally, we found that the abundance and activation status of tumor-infiltrating CD8^+^ T cells were restored by 4-PBA, which might be ascribable to attenuated suppressive capacity of MDSCs by reduced ER stress (Figure 6D-G). On the contrary, tumor-infiltrating CD4^+^ T cells showed no significant differences regardless of treatment. Taken together, these results show that 4-PBA restored anti-tumor immunity by dampening the ER stress-exacerbated suppressive capacity of MDSCs.

## DISCUSSION

In the current study, ER stress induced by long-term Tg treatment of tumor-bearing mice accelerated tumor growth, as compared with that of vehicle-treated mice. Relief of ER stress by 4-PBA reduced the tumor growth to the level observed in non-treated mice. The tumor microenvironment leads to hypoxia, glucose and amino acid insufficiency, and ER biosynthesis malfunctioning [14, 22]. Thus, there must be an elevation of ER stress and the unfolded protein response within tumor cells, and tumor-infiltrating cells, including MDSCs, might also be under ER stress. However, because 4-PBA alone could not induce tumor regression (Supplementary Figure 6), we concluded that tumor tissue may possess intrinsic mechanisms to cope with ER stress in hypoxic conditions, which mimic the effect of 4-PBA.

How systemic ER stress affects MDSC *in vivo*? Enhanced ER stress by Tg treatment increased the number and the suppressive function of MDSCs whereas reduced ER stress by 4-PBA treatment significantly reduced them (Fig 4D-E and supplement Fig 3G). However, Tg treatment did not increase the proliferation of CT26 tumor cells *in vitro* (Fig 1F). Therefore, we expected that ER stress might be able to directly stimulate MDSCs expansion and enforce their suppressive activity. On the other hand, the larger tumor had more hypoxic condition inside the mass than smaller tumor, and consequently can influence on the further expansion of MDSCs *in vivo*. Our results showed that ER stress by Tg did not increase the proliferation of CT26 cells consistent with several other reports (Figure 1F). However, recent studies suggested that ER stress can induce GSK-3β activation [23], and their activation plays an important role in the proliferation of human ovarian and colorectal cancer cells [24, 25]. Therefore, we could not rule out the possibility that enhanced ER stress could make tumor growth faster, which could increase the proliferation and suppressive function of MDSCs.

The immunosuppressive function of MDSCs has been shown to be dependent on several mechanisms, such as arginine and cysteine metabolism and reactive oxygen species generation [18, 22]. The expression of iNOS and ARG1 in MDSCs has been shown to be especially critical for the suppression of T cell function in an antigen-independent manner [15, 26]. In addition, HIF-1α induced under hypoxic conditions was involved in the up-regulation of iNOS and ARG1 in MDSCs [22]. HIF-1α induced proangiogenic factors such as VEGFR1, PDGF-B, and angiopoietins, and HIF-1α expression in MDSCs, was associated with immunosuppressive function and tumor progression [22, 27]. In contrast, splenic MDSCs, which have high levels of NOX2, increased production of reactive oxygen species, but failed to suppress antigen-independent T cell activation [22]. In addition, differences have been reported in the compositions of MDSC subsets in the spleen and in tumors [28, 29]. Granulocytic MDSCs in the spleen produce high levels of ROS via NOX2 [18], whereas up-regulated ARG1 and iNOS in tumor-infiltrating MDSCs was reported to be critical for their suppressive function [17]. In the current study, we found that the level of NOX2 was much higher in splenic MDSCs than in tumor-infiltrating MDSCs. However, levels of ARG1 and iNOS were significantly increased by Tg treatment in tumor-infiltrating MDSCs, but not in splenic MDSCs. These results suggest that the expression of ARG1 and iNOS in MDSCs is critical for their suppressive function, and could be increased by Tg-mediated ER stress under hypoxic conditions within tumor tissue, because in MDSCs their expression was correlated with immunosuppressive capacity against CD4^+^ T cells. Recently, we also showed that Myd88, an adaptor molecule critical for the activation of ARG1 and NOX2 expression, was critical for the immunosuppressive function of MDSCs [6].

The induction of ER stress in cancer cells by oxaliplatin or anthracyclines has been shown to induce calreticulin (CRT) localization outside the cell membrane, which mediates immunogenic apoptosis to stimulate tumor-specific CD8^+^ T cell responses [30]. Tg treatment in mouse embryonic fibroblast (MEF) and neuroblastoma cells stimulated CRT cell surface exposure through ER Ca^2+^ depletion, and also promoted phagocytosis of apoptotic bodies [1]. However, we could not find any evidence of increased antitumor immune response caused by systemic administration of Tg. Instead, Tg treatment accelerated tumor growth as compared to vehicle treatment. Thus, ER Ca^2+^ depletion induced by Tg might not be sufficient to induce antigen presentation, even though phagocytosis of CRT-exposing apoptotic cancer cells was increased. To efficiently induce a tumor-specific CD8^+^ T cell response, an antigen needs to be presented by MHC class I molecules after phagocytosis [5]. However, this process could be hampered by multiple prerequisite steps for successful antigen presentation. Most importantly, lysosomal degradation must occur for antigen loading into the MHC class I pocket. Degradation of intracellular protein or organelles via lysosomal fusion during autophagy could increase lysosomal antigen degradation for MHC class I presentation [31]. Erp57, an ER-resident thiol disulfide oxidoreductase that forms a complex with CRT, was recently reported to modulate STAT3 signaling [32]. Because STAT3 activity is negatively regulated by Erp57 and dependent on the formation of Erp57-CRT complexes [32], Tg-induced ER stress might induce Erp57 malfunction by disassociating it with CRT. A further explanation for enhanced Tg-mediated tumor growth is its known function as a tumor promoter in certain circumstances *via* the Src-MAP kinase pathway [33], and PI-3 kinase-mediated activation of the serine/threonine protein kinase protein kinase B (PKB) by Tg treatment may enhance survival and proliferation of some cell types [34].

4-PBA has been used for the treatment of urea cycle disorders in children because of its function as an ammonia scavenger [35]. In addition, 4-PBA has also been studied as a potential treatment for several cancers, including human solid malignant tumors and recurrent malignant gliomas [36]. The inhibition of histone deacetylation by 4-PBA can repress gene expression, including that of genes related to tumor suppression, and represents an alternative treatment for cystic fibrosis [37]. The histone deacetylation inhibition activity of 4-PBA also resulted in decreased cancer proliferation [36]. More importantly, the chemical chaperone function of 4-PBA, which mimics intracellular protein chaperones and promotes protein folding in the ER, has been suggested as a means to treat pathological conditions related to several abnormal protein localization and aggregation disorders, such as Alzheimer's disease [38], amyotrophic lateral sclerosis [39], and lysosomal storage diseases. Indeed, recent studies showed that ER stress attenuation by 4-PBA restored glucose homeostasis in a mouse model of type 2 diabetes and alleviated colitis in mice [40, 41]. Although in this study we focused on the function of 4-PBA as a chemical chaperone to facilitate the correction of unfolded and misfolded proteins in the ER, tumor regression could be partially mediated by the inhibitory effect of 4-PBA on histone deacetylation [42]. Thus, the effect of butyrate on tumor growth after Tg treatment should be assessed, because it does not possess chemical chaperone activity, but can induce histone hyperacetylation by inhibiting histone deacetylation [43].

Several cytokines secreted by MDSCs inhibit the function of CTLs [44]. Accordingly, we found that Tg-mediated ER stress decreased the number and the activation of tumor-infiltrating CD8^+^ T cells, and 4-PBA treatment restored these parameters (Figure 6E and 6G) although the number and the activity of CD4^+^ T cell and CD8^+^ T cells in the spleen and lymph node were not affected by ER stress (Figure 2). Therefore, we suggest that ER stress reduction by 4-PBA treatment could overcome the immunosuppressive environment produced by MDSCs, particularly in tumors with excessive ER stress induced by Tg treatment, to restore anti-tumor immunity nearby tumor mass.

Recent studies showed that ER stress induced by Tg in prostate cancer cells up-regulated lipocalin 2 in an NF-κB-dependent manner, because ER stress inhibition by 4-PBA and NF-κB inhibition by BAY11-7082 inhibited lipocalin 2 induction [16]. Thus, combination of 4-PBA with NF-κB inhibitors such as MG132 and BAY11-7082 might reduce ER stress-mediated tumor progression in our model. As another possible way to reduce ER stress, we and others have examined the consequences of activation of autophagy [45, 46]. Autophagy, especially macroautophagy, is a lysosomal-dependent degradation pathway that removes unnecessary cytosolic proteins and organelles, and is activated, at least partly, for the resolution of ER stress [47]. Several methods of autophagy activation, including treatment with rapamycin, salubrinal, and α-mangostin, represent candidates for ER stress reduction in cancer models *via* repression of the immunosuppressive function of MDSCs [45, 48, 49]. In addition, lithium chloride, a GSK-3β inhibitor, could attenuate Tg-mediated enhanced tumor growth, because Tg-induced apoptosis was prevented by GSK-3β inhibition [23].

Collectively, in our study, enhanced ER stress in tumor-bearing hosts increased tumor growth. Acceleration of tumor growth by ER stress was mediated by modulating the levels of ARG1 and iNOS in tumor-infiltrating MDSCs, which enhanced their immunosuppressive function. Therefore, ER stress reducers such as 4-PBA could restore anti-tumor immunity by dampening immunosuppressive MDSCs that are exacerbated by ER stress.

## MATERIALS AND METHODS

### Mice

All experiments were approved by the Institutional Animal Care and Use Committee of Kangwon National University. Wild-type BALB/c and C57BL/6 mice were purchased from Charles River Laboratories (Orient Bio Inc., Sungnam, Korea). OVA-specific OT-II (C57BL/6 background) TCR transgenic mice were purchased from Jackson Laboratories (Bar Harbor, ME). All mice used in the experiments were purchased at 6 weeks of age. The mice were kept in the Animal Center for Pharmaceutical Research at Kangwon National University. To establish tumors, mice were subcutaneously (*s.c.*) injected with 10^6^ tumor cells on the left flank.

### Cell line

Human HER2/*neu*-expressing CT26 cells (hHER2/CT26) [4, 5, 50] and mouse TC-1 cervical cancer cells were used as tumor cells in these studies. To establish tumors, 10^6^ HER2/CT26 or TC-1 tumor cells were injected *s.c.* into the left flank of BALB/c or C57BL/6 mice, respectively. Tumor growth was measured by calipers 3 times per week.

### Reagents and antibodies

Mice were treated with 100 μg/kg Tg (Sigma-Aldrich, St. Louis, MO) and 10 mg/kg sodium 4-phenylbutyrate (Calbiochem, San Diego, CA) every day. All antibodies used for flow cytometry analysis were purchased from BD Biosciences (San Jose, CA).

### Isolation of tissue-infiltrating MDSCs

To obtain tissue-infiltrating MDSCs, tumor tissue was gently homogenized with a MACS dissociator (Miltenyibiotec, Germany) and treated with 0.5 mg/mL collagenase type IV (Sigma-Aldrich, St. Louis, MO) for 30 min. Cells were harvested by gentle pipetting and exposed to a 40–70% percoll gradient to enrich mononuclear cells. To isolate MDSCs, cells were treated with an anti-CD11b^+^ microbead and isolated by MACS cell separation.

### Quantitative real-time PCR

Total RNA was isolated from 10^6^ MDSCs using the RNA Extraction Mini Kit (iNtRON, Korea). Reverse transcription was performed using the cDNA Synthesis Mini Kit (iNtRON), and quantitative real-time polymerase chain reaction (PCR) was conducted using THUNDERBIRD™ SYBR qPCR Mix (Toyobo, Japan). The following primers were used:

**Table:** 

iNOS	forward	5′-AGGAAGTGGGCCGAAGGAT-3′
	reverse	5′-GAAACTATGGAGCACAGCCACAT-3′
ARG1	forward	5′-AACACGGCAGTGGCTTTAACCT-3′
	reverse	5′-GTGATGCCCCAGATGGTTTTC-3′
Nox2	forward	5′-GACCCAGATGCAGGAAAGGAA-3′
	reverse	5′-TCATGGTGCACAGCAAAGTGAT-3′
BiP	forward	5′-ACTTGGGGACCACCTATTCCT-3′
	reverse	5′-ATCGCCAATCAGACGCTCC-3′
ATF4	forward	5′-ATGGCCGGCTATGGATGAT-3′
	reverse	5′-CGAAGTCAAACTCTTTCAGATCCATT-3′
Chop	forward	5′-CTGGAAGCCTGGTATGAGGAT-3′
	reverse	5′-CAGGGTCAAGAGTAGTGAAGGT-3′
Erdj4	forward	5′-TCAGAGAGATTGCAGAAGCG-3′
	reverse	5′-GACTCCCATTGCCTCTTTGT-3′
IL-6	forward	5′- CTGGAGTCACAGAAGGAGTGG-3′
	reverse	5′- GGTTTGCCGAGTAGATCTCAA-3′
CXCL1/KC	forward	5′- TGAGCTGCGCTGTCAGTGCC-3′
	reverse	5′- GCGTTCACCAGACGGTGCCA-3′
TNF-α	forward	5′- TGGGAGTAGACAAGGTACAACCC-3′
	reverse	5′- CATCTTCTCAAAATTCGAGTGACAA-3′
β-actin	forward	5′-CCTAGGCACCAGGGTGTGAT-3′
	reverse	5′-TCTCCATGTCGTCCCAGTTG-3′

### Western blot

Total protein lysates from tumor cells or tissue were prepared by sonication in lysis buffer (iNtRON). Equal amounts of lysates were boiled at 100°C and resolved using 8–12% sodium dodecyl sulfate polyacrylamide gel electrophoresis (SDS-PAGE). Proteins were transferred to polyvinylidene difluoride (PVDF) membranes (Millipore, Billerica, MA, USA) that were blocked with 5% milk in tris-buffered saline and Tween 20 and incubated overnight with the primary antibody, and proteins were detected with an horseradish peroxidase (HRP)-conjugated antibody (Cell Signaling Technology). Membranes were developed by the enhanced chemiluminescence (ECL) method using femtoLUCENT™ PLUS-HRP (G-Biosciences, St. Louis, MO, USA)

### *In vitro* MDSC suppression

OVA-specific TCR transgenic OT-II CD4^+^ T cells (10^5^/well) isolated from the spleens of OT-II mice were labeled with 1 μM CFSE, stimulated with H-2^b^-restricted OVA MHC class II epitope peptide (ISQAVHAAHAEINEAGR)-pulsed splenic DCs (10^3^/well), and cocultured with MDSCs (2 × 10^5^/well) for 72 h. CFSE dilutions of OT-II CD4^+^ T cells were analyzed using flow cytometry.

### *In vivo* cytotoxicity assay

The *in vivo* cytotoxicity of CD8^+^ T cells was assessed as described previously [6, 51]. Briefly, syngeneic splenocytes and lymph node (LN) cells were divided into 2 fractions of equal number that were either loaded with 1 μg/mL of cytotoxic T lymphocyte (CTL) epitope peptide (human HER2/*neu* p63 [TYLPTNASL]) or left unpulsed. Peptide-pulsed cells were labeled with 10 μM CFSE (Invitrogen, Carlsbad, CA) and unpulsed cells were labeled with 1 μM CFSE. Equal numbers of CFSE^high^ and CFSE^low^ cells were mixed and injected intravenously into mice. After 24 h, the splenocytes from treated mice were analyzed to assess antigenic peptide-specific target lysis. The specific lysis was calculated as follows:

$$\begin{array}{l} \text{r }(\text{ratio})=(\%CFSE^{low}/\%CFSE^{high})\\ \%\text{ lysis}=(1-[r_{unprinted}/r_{printed}])\times 100 \end{array}$$

### Determination of cell viability

Cell viability was determined using the Dojindo Cell Counting Kit-8 (Dojindo, Gaithersburg, MD, USA) according to the manufacturer's instructions. Splenocytes and tumor cells were seeded in 96-well plates and allowed to adhere overnight. The cells were treated with Tg at 37°C for 24 h. The absorbance at 450 nm was measured with a SPECTRA MAX 340 instrument (Molecular Devices, US).

### Statistical analysis

Student's t-test was used to compare differences between the 2 groups. To compare multiple groups, we performed one-way ANOVA followed by Tukey's post hoc test. Values of *p* < 0.05 were considered to be significant.

## SUPPLEMENTARY FIGURES


