# Supplementary material for: Elevated endoplasmic reticulum stress reinforced immunosuppression in the tumor microenvironment via myeloid-derived suppressor cells
Source: Oncotarget. 2014 Dec 2;5(23):12331–45. doi: 10.18632/oncotarget.2589 (PMC4322987; doi:10.18632/oncotarget.2589)
Supplement: Supplementary file 1 [file oncotarget-05-12331-s001.pdf]

SUPPLEMENTARY FIGURES

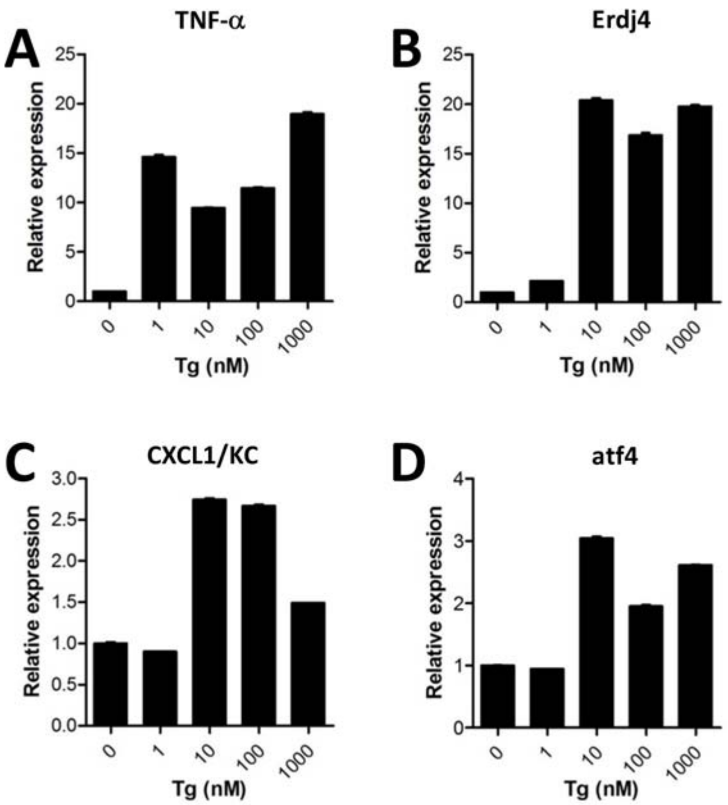

Supplementary Figure S1: Tg-induced ER stress in HER2/CT26 cells. HER2/CT26 cells were cultured with Tg for 24 h and mRNA levels of ER stress genes were measured by RT-qPCR. **(A-D)** mRNA levels of *TNF-α*, *Erdj4*, *CXCL1*, and *atf4* in Tg-treated HER2/CT26 cells.

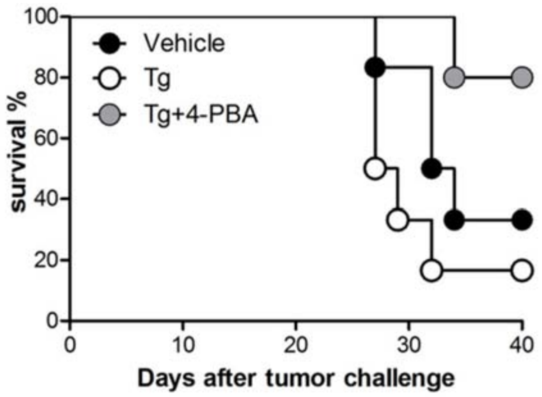

Supplementary Figure S2: 4-PBA increased the survival of tumor-bearing mice caused by Tg treatment. Daily treatments with 100 µg/kg Tg or 10 mg/kg 4-PBA were initiated 2 weeks after *s.c.* injection of TC-1 tumor cells into C57BL/6 mice (n = 6).

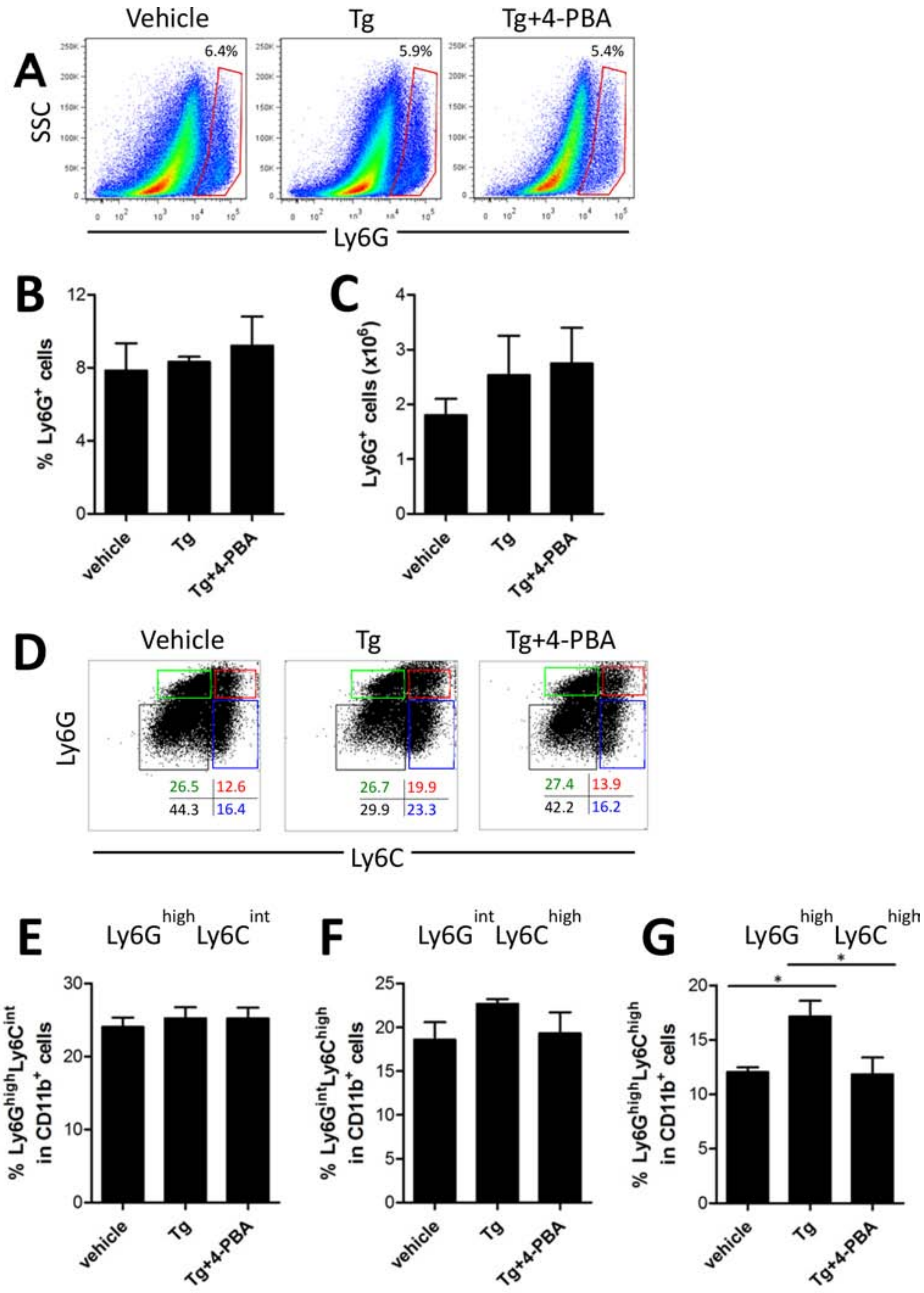

Supplementary Figure S3: The populations of tumor-infiltrating MDSCs were not affected by ER stress. BALB/c mice were injected *s.c.* with 2×10^5 HER2/CT26 cells. At 4 weeks after tumor inoculation, MDSCs were isolated from tumor tissues and were assessed by flow cytometry. **(A)** tumor-infiltrating $Ly6G^+$ cells. **(B)** percentages, and **(C)** absolute number, of tumor-infiltrating $Ly6G^+$cells ($n = 3$). **(D)** $Ly6G^+$ and $Ly6C^+$ subsets among $CD11b^+$ MDSCs. **(E-G)** summary graph for sub-populations in $CD11b^+$ MDSCs ($n = 3$). Graphs show mean ± SEM. $^*p < 0.05$ using *Student'*s t-test.

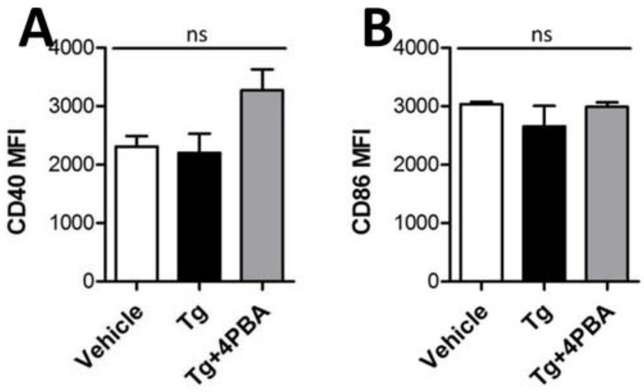

Supplementary Figure S4: Surface expression of costimulatory molecules on tumor-infiltrating MDSCs. (A-B) MDSCs were isolated from spleen tissues, and CD40 and CD86 expression was assessed by flow cytometry. ns, not significant.

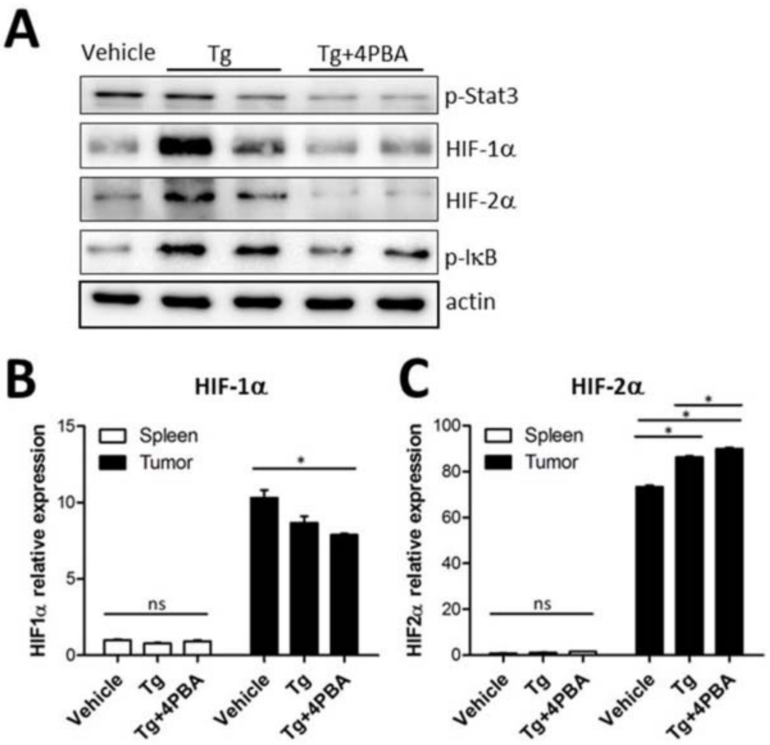

Supplementary Figure S5: 4-PBA reduced HIF expression enhanced by Tg-mediated ER stress. **(A)** Immunoblot of tumor-lysate from tumor-bearing mice treated with vehicle, Tg, and Tg with 4-PBA. **(B-C)** mRNA levels of *HIF-1α* and *HIF-2α* from spleen- or tumor-filtrating MDSCs. ns, not significant, $^{*}p < 0.05$ using one-way ANOVA.

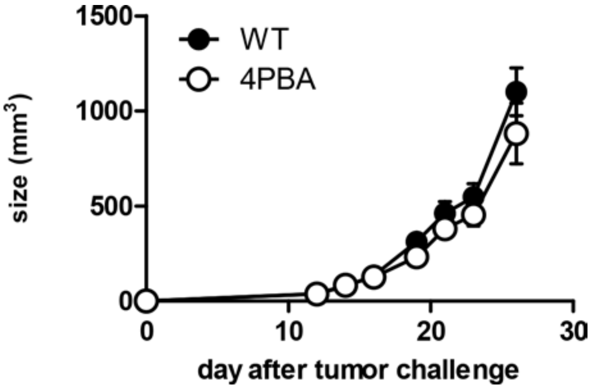

Supplementary Figure S6: 4-PBA treatment alone did not decrease tumor growth. BALB/c mice were injected *s.c.* with 10^6 HER2/CT26 cells. Daily treatments with 10 mg/kg 4-PBA were initiated 2 weeks after tumor inoculation.
